# Diversity of flux distribution in central carbon metabolism of *S. cerevisiae* strains from diverse environments

**DOI:** 10.1186/s12934-016-0456-0

**Published:** 2016-04-05

**Authors:** Thibault Nidelet, Pascale Brial, Carole Camarasa, Sylvie Dequin

**Affiliations:** SPO, INRA, SupAgro, Université de Montpellier, 34060 Montpellier, France

**Keywords:** Diversity, Flux balance analysis, Metabolic flux, Modeling, *S. cerevisiae*

## Abstract

**Background:**

*S. cerevisiae* has attracted considerable interest in recent years as a model for ecology and evolutionary biology, revealing a substantial genetic and phenotypic diversity. However, there is a lack of knowledge on the diversity of metabolic networks within this species.

**Results:**

To identify the metabolic and evolutionary constraints that shape metabolic fluxes in *S. cerevisiae,* we used a dedicated constraint-based model to predict the central carbon metabolism flux distribution of 43 strains from different ecological origins, grown in wine fermentation conditions. In analyzing these distributions, we observed a highly contrasted situation in flux variability, with quasi-constancy of the glycolysis and ethanol synthesis yield yet high flexibility of other fluxes, such as the pentose phosphate pathway and acetaldehyde production. Furthermore, these fluxes with large variability showed multimodal distributions that could be linked to strain origin, indicating a convergence between genetic origin and flux phenotype.

**Conclusions:**

Flux variability is pathway-dependent and, for some flux, a strain origin effect can be found. These data highlight the constraints shaping the yeast operative central carbon network and provide clues for the design of strategies for strain improvement.

**Electronic supplementary material:**

The online version of this article (doi:10.1186/s12934-016-0456-0) contains supplementary material, which is available to authorized users.

## Background

Cellular metabolism entails a large number of reactions that are involved in the conversion of various resources into precursors and energy for biosynthesis and cellular compounds. The rates of these reactions, i.e. fluxes, reflect metabolic activity through the operative network. Fluxes are the combined result of regulation at many different biological levels, such as transcription, translation, post-translational protein modification and protein–protein interactions. Therefore, metabolic fluxes are a global representation of the cellular phenotype expressed under specific conditions; thus, analyzing flux distribution is a valuable approach to study cell metabolism [1].

While intracellular fluxes are difficult to measure experimentally, they can be predicted by different methods that rely on constraint-based models (CBM) that formalize the metabolic network as a stoichiometry matrix. These CBM range from small networks focused on a specific aspect of cellular metabolism to genome-scale models that include all reactions of a given organism. The first step to solve these systems and predict fluxes from these networks is to add constraints on the input and output fluxes. Depending on the number of constraints and the size of the network, it is possible to estimate the fluxes in some cases; this approach is referred to as metabolic flux analysis (MFA). However, in most cases, adding constraints only on input and output data is not sufficient; therefore, there are two possibilities: the ^13^C-MFA [2] and the flux balance analysis (FBA), [3]. In the ^13^C-MFA approach, cells are fed ^13^C-labeled glucose, and the analysis of the subsequent ^13^C enrichment in different amino-acids generates experimental data that can be used to constrain internal fluxes and therefore estimate intracellular fluxes [1, 2]. By contrast, the FBA is based on the choice of an optimal solution in the space of possible solutions defined by the constraint stoichiometry matrix. This solution will optimize an objective function [3]; therefore, the predicted flux distribution depends on the objective function that is used [4–6]. Objective functions commonly used are maximization of ATP production [7], minimization of metabolic adjustment [8, 9] or, most frequently, maximization of biomass production [10, 11]. These objective functions appear to be more or less effective depending on the conditions, constraints and models, without one of them emerging in particular [6].

In a previous study, ^13^C-MFA and FBA approaches have been used to predict intracellular fluxes of central carbon metabolism of *S. cerevisiae* in conditions where the intracellular redox balance is modified [12]. Comparable relative changes between environments were obtained regardless of the predicting method, even if some flux predictions differed, in particular for the pentose phosphate pathway (PPP) [12].

Understanding how metabolic fluxes are modulated by environmental and/or genetic perturbations is a central question to understanding cellular physiology. For example, the FBA approach has been used to study the flux distribution sensitivity of *S. cerevisiae* wine yeast to environmental conditions, including various glucose concentrations, temperature or acetoin levels [9, 13]. In these studies, the PPP was one of the most variable fluxes, while the glycolytic flux remained virtually unchanged. These approaches have also been widely used to study network robustness and the effects of deletion mutants [14–16]. For example, using a ^13^C flux approach in *S. cerevisiae*, Blank et al. [17] have shown that network redundancy through duplicate genes is a major determinant of genetic network robustness (75 %), while alternative pathways contribute to a lesser extent (25 %). Using a similar approach, Velagapudi et al. [18] studied the effect of knockout strains on the rerouting of metabolic fluxes in glucose and galactose media, highlighting interesting links between pathways, such as a positive correlation between flux through the PPP and biomass yield.

Flux prediction has also been used to guide metabolic engineering and strain improvement strategies [19, 20]. For instance, Bro et al. used CBM to predict the best possible metabolic engineering strategies to increase ethanol yield [21]. Guided by a genome scale model, they developed a strain with a glycerol yield reduced by 40 % and an ethanol yield increased by 3 % without affecting growth. Other examples include the prediction of strategies to optimize the yields of purine [5], succinic acid [20, 22] or proline [23].

The estimation of metabolic fluxes was also used in a few studies to investigate the divergence of flux distribution among species. ^13^C flux analysis has been used to compare flux distributions in central carbon metabolism for pairs of species, including *S. cerevisiae* and *Phaffia rhodozyma* [24] or *S. cerevisiae* and *Pichia stipitis* [25], highlighting differences in the relative flux distribution, especially for the PPP. Using ^13^C flux analysis, Blank et al. [17] and Christen and Sauer [26] studied the diversity of flux distributions in fourteen and seven yeast species, respectively. In both studies, similar correlations were shown between metabolic pathways, in particular, a trade-off between glycolysis and TCA fluxes and a positive correlation between biomass production and flux through the PPP.

In recent years, tremendous knowledge has been gained regarding the genetic and phenotypic diversity of *S. cerevisiae* [27–34]. The phenotypic diversity in these studies has mainly been addressed by the comparison of growth rate patterns in various media. Several other studies have begun to characterize the diversity of more various phenotypic traits. Spor et al. [35] have studied the phenotypic diversity of six life-history traits and three metabolic traits of different strains of *S. cerevisiae*, and they have identified two main life-history strategies, the “ants” and “grasshoppers,” which are characterized by divergence in cell size, reproductive rate and carrying capacity. A wider phenotypic analysis, performed with 72 *S. cerevisiae* strains from different origins and studying seven life-history traits and eleven metabolic traits, showed that strain origin has a wide impact on phenotypes [36]. Other studies have focused on nitrogen availability [37] or bio-ethanol-related traits [38].

Thus, the intra-species diversity of flux distribution remains unexplored. Studying the diversity of metabolism, particularly of metabolic fluxes, is fundamental to understanding the constraints and regulations that shape strain phenotypes. The functional and regulatory properties of yeast central carbon metabolism (CCM) determine most of the phenotypic traits relevant for various industrial processes, including food and beverage production (wine, bread, beer, cheese etc.), bioethanol or the use of yeast as a cell factory. For example, the fermentation rate, ethanol yield or production of acetate, and even aroma production are all dependent on carbon metabolism.

Thus, understanding how metabolic constraints structure metabolic pathways may enable a better exploitation of this diversity for industrial biotechnology. The objective of this study was to characterize the diversity of metabolic fluxes in a large set of *S. cerevisiae* strains from different genetic and ecological origins. To this end, we used a FBA approach to predict flux distribution for 43 strains of *S. cerevisiae* from six different ecological origins: bread, rum, wine, flor, Mediterranean and American oak. The analysis of flux distribution dataset enabled us to identify the most flexible/robust fluxes and several correlations or trade-offs between metabolic pathways. In addition, we analyzed the flux structuration to strain origin in order to observe a possible convergence.

## Results

**Fig. 1:**
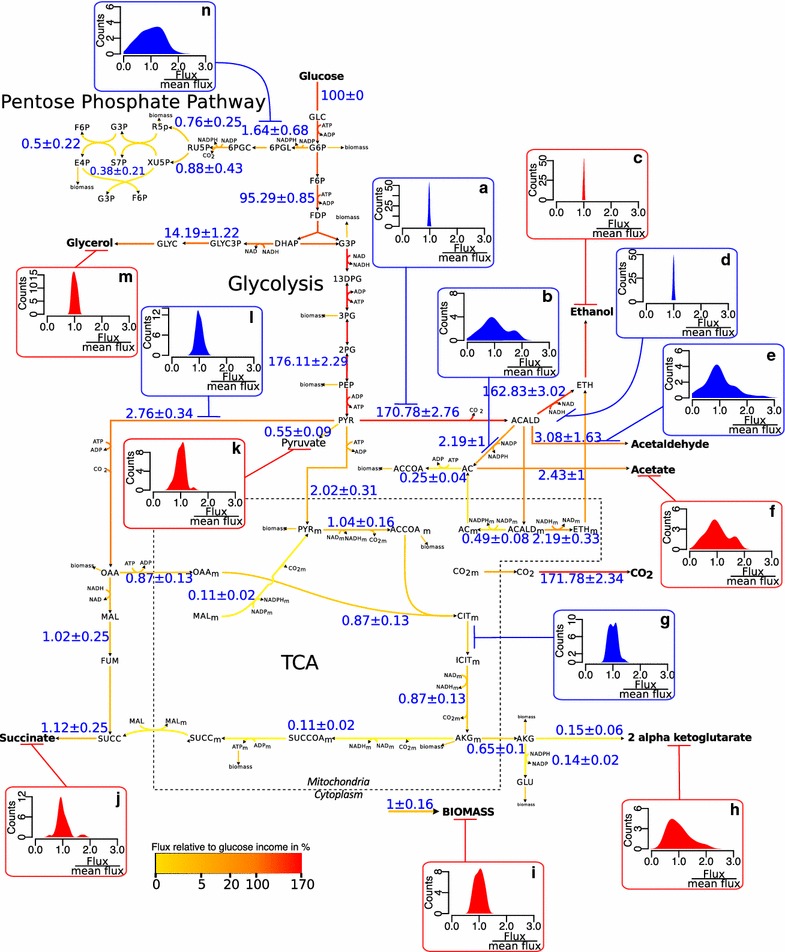
Schematic representation and distributions of fluxes in central carbon metabolism. Schematic representation of the average flux of 43 strains. The colors of the lines are representative of the average flux values across all strains expressed as a percentage of the glucose input and represented by a gradient of color from *yellow* to *red*. The average flux values ± the standard deviation are indicated by *blue* numbers for selected and representative reactions. Distribution of flux values for several selected reactions (**a**–**n**). The fluxes are normalized by the average flux of each reaction and therefore are represented by between 0 and 3, where 1 is the average flux. The reactions constrained by experimental data are indicated in *red*, and those predicted by the model are in *blue*

In this work, we used DynamoYeast, a previously developed constraint-based model of central carbon metabolism [9], to study the diversity of metabolic flux distributions for 43 strains of six different ecological origins: “Bread,” “Rum,” “Wine,” “Flor,” “Mediterranean Oak” (Med_Oak) and “American Oak” (Oak). This model comprises the cytosol, mitochondria and extracellular medium and includes upper and lower glycolysis, the PPP, the synthesis of glycerol, the synthesis of ethanol, and the reductive and oxidative branches of the TCA as the main metabolic pathways (Fig. 1).

Fermentation was performed for all strains in a synthetic medium simulating grape must, containing high sugar and low nitrogen concentrations. Typical wine fermentation comprises a lag phase, a growth phase of approximately 24–36 h followed by a stationary phase, during which most of the sugar is fermented (reviewed in Marsit and Dequin [39]). We measured the production of biomass and metabolites, including ethanol, glycerol, acetate, succinate, pyruvate and alpha-ketoglutarate during the growth phase (at 11 g/L CO_2_ released), which can be considered as steady state (a prerequisite to CBM). These experimental data (±2.5 %) were used to constrain the model as upper and lower bound to then perform a flux balance analysis (FBA).

The FBA consists of choosing the best solution for the objective function in the space of possible fluxes. Instead of using an optimization that maximizes biomass flux, which is frequently used in FBA studies, we chose to minimize the glucose input, allowing us to use the experimental biomass as a constraint for the model. By making this optimization choice, we considered that the yeasts were optimal, in that they used the least amount of resources (here the glucose input) to produce biomass and fermentation byproducts. This strategy also has the advantage of optimizing the modeling approach by maximizing the use of available experimental data. Using this approach, we obtained a flux distribution for 68 fluxes of the central carbon metabolism for each strain, expressed as relative fluxes normalized to the specific glucose uptake in the corresponding strain.

In this type of optimization, the given solution is often not the only one that meets the optimization criterion; i.e., different possible pathways are perfectly equivalent for the optimization criteria. We thus decided to characterize all equivalent solutions to determine the fluxes that varied most between alternative solutions, which would therefore correspond to poorly predicted fluxes. To achieve this, we first fixed the input and output fluxes to the exact values predicted by the FBA, and we then used the “enumerateOptimalSolution” algorithm from the cobra toolbox [40] to identify all alternative solutions. For the large majority of fluxes, we found only one predicted value, except for the fluxes of the reductive branch of the TCA involved in the conversion of malate to fumarate and then to succinate, for which two solutions were identified. Indeed, these fluxes can be cytoplasmic or mitochondrial, which had no effect on the other fluxes predicted by the model, as the transport between these two compartments of the metabolites was free in our model. Setting either option to zero suppressed the alternative solution. We finally retained the solution going through the cytoplasm, which involved fewer reactions (no mitochondrial transport).

**Fig. 2:**
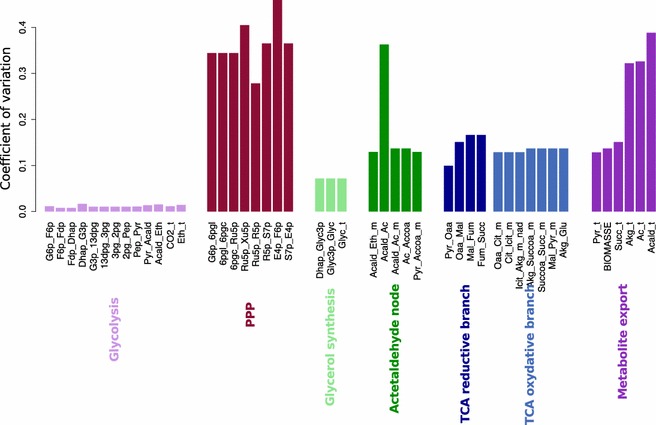
Coefficient of variation for the model’s fluxes. The coefficient of variation (ratio of the standard deviation to the mean) of each *flux* is represented as a *vertical bar*. The *vertical bars* are ordered by metabolic pathways: glycolysis and ethanol synthesis (*pink*), PPP (*dark red*), glycerol synthesis (*light green*), acetaldehyde node (*green*), reductive branch of the TCA (*dark blue*), oxidative branch of the TCA (*blue*) and output fluxes (*purple*)

Then, we considered the biological variance between strains to identify the more robust and variable fluxes of the central carbon metabolism by studying the individual flux distributions (Fig. 1) and by comparing the variation coefficients (the ratio of the standard deviation to the mean) between fluxes (Fig. 2). Substantial differences were found in the variability of fluxes depending on the metabolite pathways (Fig. 2). The glycolysis and ethanol synthesis pathways displayed almost no variation (e.g. Pyr_Acald: 170.78 ± 2.76 %, Fig. 1a; Acald_Eth: 162.83 ± 3.02 %, Fig. 1d). The reductive and oxidative branches of the TCA (e.g. Cit_Icit_m: 1.02 ± 0.24 %, Fig. 1g; Pyr_Oaa: 2.76 ± 0.34 %, Fig. 1l), the glycerol synthesis pathway (e.g. Glyc_t: 14.41 ± 1.29, Fig. 1m) and the biomass synthesis (BIOMASS: 1.02 ± 0.18 %, Fig. 1i) displayed a moderate variation. By contrast, the PPP pathway was the highest variable pathway (e.g. G6p_6pgl: 1.64 ± 0.68 %, Fig. 1n).

The acetaldehyde node displayed a particular pattern as it includes individual fluxes with very different variabilities (Fig. 2): besides the invariant synthesis of ethanol, the synthesis of acetate was highly variable with a wide bimodal distribution (Acald_Ac: 2.19 ± 1 %, Fig. 1b). The acetate output (Ac_t: 2.43 ± 1 %, Fig. 1f) and the excretion of acetaldehyde (Acald_t: 3.08 ± 1.63 %, Fig. 1e) were also highly variable.

**Fig. 3:**
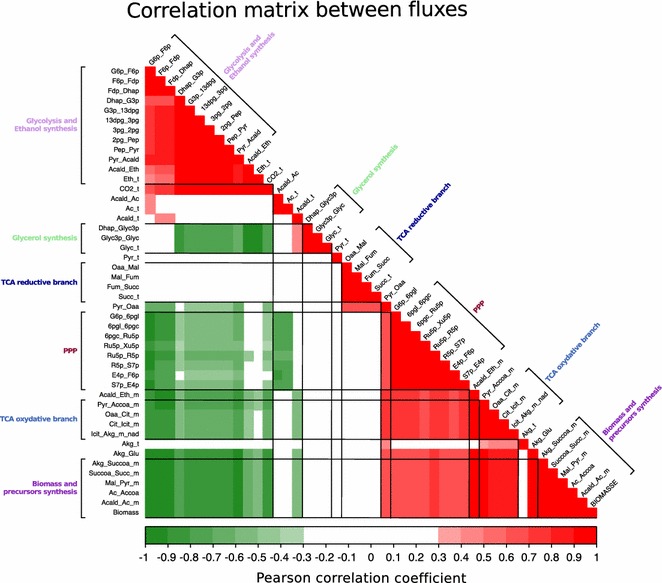
Correlation matrix. Matrix of correlations between the model’s fluxes. The Pearson correlation values between each pair of fluxes are represented as a gradient of colors from *green* (−1) to *red* (+1). The fluxes are ordered by metabolic pathways

Then, we searched for potential links between fluxes by studying all correlations between the model’s fluxes (Fig. 3). This approach first highlighted a “pathway block” structure, where fluxes were highly correlated to each other and operated almost like a single flux. For example, all the fluxes of the PPP displayed a Pearson correlation coefficient between them greater than 0.985 (Fig. 3). We identified seven blocks: upper glycolysis, lower glycolysis, glycerol synthesis, the TCA reductive branch, the PPP, the TCA oxidative branch and the biomass block. The latter included the biomass synthesis reaction and all the fluxes that were only used to produce one of the biomass precursors. For example, cytoplasmic acetyl-CoA was only used in the model as a precursor of biomass (because the model never predicted its mitochondrial transport). Thus, the flux of acetyl-CoA synthesis (Ac_Accoa) was perfectly correlated with biomass synthesis (Fig. 3).

**Fig. 4:**
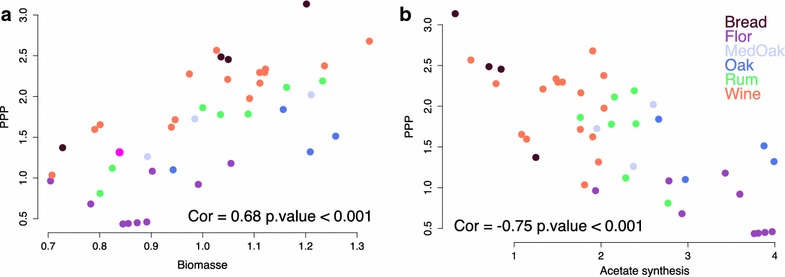
Relationship between fluxes through the PPP and the biomass flux or the acetate synthesis flux. Relationship between the G6P_6Pgl flux representative of PPP and biomass flux (**a**). Relationship between the G6P_6Pgl flux representative of PPP and the flux of acetate synthesis (Acald_Ac) (**b**). Each strain is represented as *dots*, with the color corresponding to the strain’s origin. The Pearson correlation values are indicated at the bottom of each *graph* as the significance of the correlation

We also found correlations between blocks that had two main origins. In first case, these correlations were compulsory due to the model structure. For example, there was an expected negative correlation between the glycerol fluxes and the lower part of glycolysis because these two pathways diverged from the upper part of glycolysis. For the same reason, the flux through the PPP was negatively correlated with upper glycolysis. Positive correlations were also found between the PPP (Fig. 4a), the TCA oxidative branch and the biomass block, which could be connected to the synthesis of biomass precursors, such as Erythrose-4-phosphate (E4P), Ribose-5-phosphate (R5p) and alpha-ketoglutarate (AKG). Other correlations were independent of the network structure and emerged from the biological data. For example, a correlation was found between the fluxes through PPP and acetate synthesis (Acald_Ac, Fig. 4b). This strong negative correlation was identified using the whole strain data set (r = −0.76, Fig. 4b). This trade-off could be linked to the synthesis of NADPH that can be achieved by these two pathways. Approximately 60 % of the NADPH demand is supplied by the PPP, but this proportion varied between 95.7 and 18.8 % depending on the strains, independently of the total production (Additional file 1: Figure S1). It is interesting to note that this trade-off did not appear in the model’s null space of possible fluxes, which indicates that this correlation is independent of the network matrix and is purely biological.

**Fig. 5:**
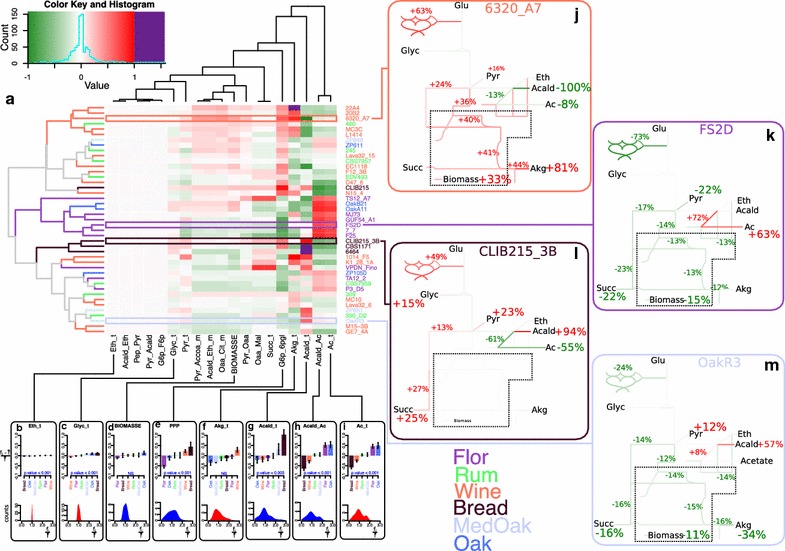
Clustering of flux deviations. Matrix of deviation from the average for 19 fluxes and all strains (**a**). Each rectangle of the matrix represents a relative deviation index calculated by dividing the deviation between the flux of one reaction for one strain and the average flux for all strains by the average flux of the corresponding reaction. Each *line* corresponds to all relative deviation indexes for one strain. Each *column* corresponds to the relative deviation indexes for one reaction and all strains. The lines and column are ordered with respect to the function of their Euclidian distances, which are represented by *dendrograms* both at the *top* and the *left* of the matrix. The distribution of all the relative deviation indexes as well as the corresponding color gradient are in the *top left* of the matrix. The *sub-graphs* represent the effect of strain origin on the relative deviation index as well as the distribution of the corresponding flux for eight selected fluxes (*red* distribution for fluxes constrained by experimental data, and *blue* for fluxes only predicted by the model) (**b**–**i**). Simplified schematic representation of the metabolic network (**j**–**m**). The relative deviation index for four selected strains of different origins is indicated as a percentage. Only the deviations greater than ±8 % are provided

Because the fluxes were mostly organized in blocks (Fig. 3), we decided to use only a subset of fluxes containing one representative flux for each block for further analysis. With this subset of 19 fluxes, we studied the deviation of each strain from the average for each flux. Then, we used a clustering method to classify the strains and fluxes as a function of their Euclidean distance (Fig. 5a). The fluxes that best separated strains were the most variable and also had binomial distributions, indicating very different behaviors across strains (Fig. 5b–i). The fluxes of acetate synthesis (Fig. 5h) and output (Fig. 5i) could separate one particular cluster of eight strains that was mainly characterized by a high production of acetate and a small flux through the PPP. The strain FS2D (Fig. 5k) of this cluster had a small flux through the PPP (−73 %), a small flux through both the TCA branch (−13 and −23 %) and small production of biomass (−15 %) but a high acetate synthesis and output (+72 and +63 %). Similarly, the flux of acetaldehyde output predicted by the model highlighted a cluster of three strains characterized by a very high production of acetaldehyde, of which Clib215_3B strain was a good example (Fig. 5l). This strain was mainly characterized by a high acetaldehyde output (+94 %), a high reductive branch of TCA (+27 %) and succinate output (+25 %), high glycerol output (+15 %) and a small acetate production and output (−61 and −55 %). The other fluxes did not allow such a clear separation of strains but illustrated small differences in similar global distributions.

Interestingly, these two particular clusters were overwhelmingly composed of strains having one ecological origin. The cluster characterized by a high production and output of acetate was composed of “Flor” strains, and the cluster with high acetaldehyde production was only composed of “Bread” strains. To better understand the effect of strain origin on flux distribution, we considered the mean fluxes by origin (Fig. 5b–i). The acetate synthesis and output fluxes (Fig. 5h, i) were approximately 50 % higher for the “Flor” and “American Oak” (Oak) strains and approximately 50 and 25 % lower for the Bread and Wine strains, respectively. This dichotomous behavior explaining the bimodal distribution of these two fluxes also presented a significant effect of the ecological origin (p < 0.001 for both fluxes). Similarly, the very long tail in the flux distribution of acetaldehyde output (Acald_t) can be explained by the “Bread” strains that produce approximately 100 % more acetaldehyde that other strains (Fig. 5g, p = 0.003). Flux through the PPP (Fig. 5e, p < 0.001) and glycerol synthesis (Fig. 5c, p < 0.001) also presented significant effects of strain origin while having less variability. By contrast, fluxes with high variability and that well separated strains, such as the alpha-ketoglutarate output (Fig. 5f), presented no significant effect of strain origin. Thus, there was no link between the extent of flux distribution and its contribution to strain origin separation.

**Fig. 6:**
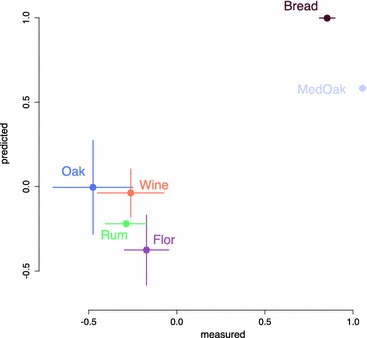
Comparison between predicted and measured acetaldehyde production. Graphical comparison of the acetaldehyde production deviation from the average calculated for each origin group between predicted (*y-axis*) and measured data (*x-axis*). The *vertical* and *horizontal bars* represent the standard errors

Thus, this analysis indicated interesting physiological differences between strains, some of which were related to the ecological origin. To experimentally confirm the higher production of acetaldehyde by the bread strains, we a posteriori measured the production of acetaldehyde for seventeen strains from various origins and compared the relative variations of production with flux prediction (Fig. 6). These experimental data confirmed our predictions, with the “Bread” strains producing 137.78 ± 5.68 mg L^−1^ of acetaldehyde on average, while the strains from other origins produced 59.88 ± 35.51 mg L^−1^ (p value < 0.001) at the fermentation time point of 11 g L^−1^ of CO_2_ produced.

Moreover, a correlation was also found within groups of strains with similar ecological origins (Fig. 4) as well as for the proportion of the NADPH demand provided by the PPP or acetate synthesis. Indeed, the “Bread” and “Wine” strains mainly produced their NAPDH by the PPP (approximately 84 and 72 %, respectively), while the six strains that predominantly produced NAPDH by acetate synthesis were “Flor” strains, with only approximately 20 % of the NADPH demand produced by the PPP (Additional file 1: Figure S1).

**Fig. 7:**
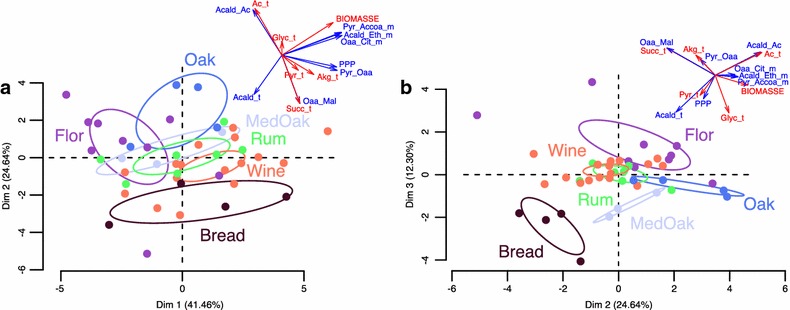
Principal component analysis of the model’s fluxes. Graphical representation of strain fluxes projected on the two plans defined by the three first axes of the PCA calculated from 14 predicted fluxes for 43 strains. The strains are represented as *dots* colored by the function of strain origin. On *top* of each *graph* is the *circle* of variables. The *red lines* correspond to constrained fluxes and the *blue lines* to predicted fluxes. Plan defined by axis 1 and 2 of the PCA (**a**). Plan defined by axis 2 and 3 of the PCA (**b**)

Finally, to obtain an integrated vision of flux structuration, we performed a principal component analysis (PCA). For this, we selected the same subset of 19 fluxes, among which we excluded the fluxes of glycolysis and ethanol synthesis on the basis that they were stronger but also less variable fluxes, which would therefore give them too much importance in the PCA. A final subset of 14 fluxes was used to perform the PCA (Fig. 7). The first three axes of the PCA explained 41.46, 24.62 and 12.3 % of the variance. The PCA plan defined by the second and third axes was the one that better separated the strains according to their origins. The second axis significantly separated the “Bread” (+2.37) and the “Oak” (−2.4) strains, and the third axis significantly separated the “Flor” (+1.84), the “Wine” (+0.67), the “Med_oak” (−0.97) and the “Bread” (−1.95) strains. The “Bread” strains at the bottom left of this PCA plan were characterized by a high production of acetaldehyde and a small production of acetate. The oak strains (“Med_oak” and “Oak”) in the bottom right had high production of glycerol and small production of succinate. The “Flor” group at the top right had high production of acetate, a small flux through the PPP and small production of acetaldehyde. This group was almost symmetrically opposed to the “Bread” group. The two remaining groups, “Rum” and “Wine,” were more central and better separated by the plan determined by the two first axes of the PCA. Finally, it is interesting to highlight that the fluxes structuring the axis were in the same proportion predicted by the model and constrained by the experimental data.

## Discussion

In this work, we used a constraint-based model of yeast fermentative central carbon metabolism to study the diversity of flux distribution among 43 strains of different origins. We used a whole set of experimental data (ethanol, glycerol, succinate, acetate, pyruvate, alpha-ketoglutarate and biomass production) to constrain the model and a FBA approach with minimization of the glucose input to predict the distribution of metabolic fluxes. This method allowed us to optimize the modeling process by using all the available biological information. We first considered the variability of the predictions to determine the confidence of the estimates. Considering alternate optimal solutions led us to conclude that the DynamoYeast model was very well determined, with only small variations in the reductive branch of the TCA due to free mitochondrial transport of the involved metabolites (malate, fumarate and succinate). This very low level of variability between alternate optimal solutions for a given set of constraints was the main advantage of using a reduced model. Indeed, the same constraints used with a genome-scale model (6th version of the consensus model, [41]) led to predicted flux distribution predictions with many alternative solutions, some of which were biologically irrelevant (data not shown).

The main objective of this study was to characterize the variability of flux distributions between *S. cerevisiae* strains from different origins. We found that this variability was strongly pathway-dependent. The glycolysis and ethanol synthesis pathways, despite being the stronger fluxes, showed almost no variability between strains. In contrast, flux through the PPP was the most variable, with a coefficient of variation more than two times higher than that of other pathways. This high variability of the PPP is in accordance with a previous study stressing high variability of the specific activity of the first enzyme of the PPP, glucose-6-phosphate dehydrogenase, in eleven *S. cerevisiae* strains [42]. This, in addition to the finding that the PPP was one of the most variable fluxes in different environments [13], suggests high flexibility of this pathway depending on environmental and genetic factors.

Our study also highlighted several correlations between metabolic pathways. The PPP produces around 2/3 of the NAPDH demand and displays a strong trade-off with the cytoplasmic synthesis of acetate from acetaldehyde (Acald_Ac in our model), the other main reaction generating NAPDH. An indication of a link between these two pathways was found in previous studies. For example, in a study comparing the flux distributions of *S. cerevisiae* during respiro-fermentative growth in different conditions of pH and NaCl concentration, Heyland et al. [43] found an inverse variation between the fluxes through acetate production and PPP, unfortunately with too few points to test for a significant correlation. Predicted fluxes between an evolved strain of *S. cerevisiae* and its ancestor showed a similar trade-off: an increased flux thought the PPP and a decreased production of acetate in the evolved strain [44].

Interestingly, among the intra-species correlations that we identified in this study, some have also previously been found when different yeast species were compared. The positive correlation between PPP and biomass fluxes (which we linked to biomass precursor synthesis) was also found in a comparative ^13^C-flux analysis of seven yeast species [26] and of fourteen other hemiascomycetous yeasts [17]. Between these fourteen hemiascomycetous, the proportion of NAPDH demand produced by the PPP varied between 60 % for *S. cerevisiae* and 90 % for *P. angusta* [17]. Similarly, in our work, the mean percentage of NAPDH produced by the PPP was 59 % (Additional file 1: Figure S1). A higher level of flux through the PPP was found for *S. cerevisiae* in the Blank study compared to this work (10 versus 2 %); this discrepancy between fluxes predicted by ^13^C-MFA or FBA is common [12]. Another correlation found in our work as in other studies was the negative correlation between glycolysis and the TCA fluxes, which have been associated with a down regulation of glycolytic genes [43].

Another issue addressed in this study is the contribution of strain origin to intra-species metabolic diversity. For the variable fluxes, the flux distribution was divergent in broadness and could also be mono-, bi- or multimodal, indicating dichotomous behavior between strains. We could explain these different patterns of distribution by strain origin peculiarities. For example, the long tail of the acetaldehyde output distribution can be explained by the four “Bread” strains that produce twice as much acetaldehyde (Fig. 5g) and the bimodal distribution of the production and output of acetate by the contrasted behavior of the “Flor” and “Bread” strains. Further, using the predicted fluxes rather than only the experimental data helps to distinguish the strains according to their origins (Additional file 1: Figure S2). Indeed, among the five fluxes (G6p_6pgl, Acald_t, Akg_t, Acald_Ac, Ac_t) that best distinguished strains from each other (especially the “Bread” and “Flor” strains), two were only accessible by the model (G6p_6pgl, Acald_t), which highlights the potential of the flux analysis approach. Interestingly, some fluxes, such as flux through the PPP, were by themselves able to separate strains by origin.

Such knowledge on the most flexible fluxes and strain-dependent flux variability could be very useful for metabolic engineering strategies aimed at rerouting metabolic fluxes. Numerous studies [44–54] have attempted to modify yeast flux distributions using metabolic or evolutionary engineering approaches or hybridization to exploit natural diversity for various biotechnological applications. Our study shows almost no diversity in the flux distributions of glycolysis or ethanol synthesis, suggesting strong constraints on these fluxes, either evolutionary or metabolic. By contrast, the fluxes through glycerol synthesis [54–57] or the PPP [42, 44] were more flexible, which makes them more interesting targets to redirect metabolic fluxes. In addition, the availability of strain-specific maps of metabolic flux distribution will provide a framework for the selection of the most relevant strains for metabolic engineering strategies.

## Conclusion

Overall, this work highlights the potential of flux analysis to identify the most variable and robust nodes of central carbon metabolism within a species and to provide information on the metabolic or evolutionary constraints that shape flux distribution. This knowledge will help to identify relevant targets and yeast strains for metabolic engineering. In addition, the availability of whole genome sequences for the strains used in this study offers a framework to decipher the links between flux distribution and strain genotypes. In particular, the finding of a strain origin effect on the distribution of various fluxes opens the way for flux quantitative trait loci (QTL) detection (fQTL) to elucidate the genetic basis of flux distribution.

## Methods

### Strains and culture conditions

The 43 *S. cerevisiae* strains of six different ecological origins (4 “Bread,” 7 “Rum,” 16 “Wine,” 9 “Flor,” 3 “Medoak” and 4 “Oak”) used in this study are listed in Additional file 2: Table S1. These strains were conserved at −80 °C and transferred to YPD agar plates 48 h before fermentation. Initial cultures (12 h, in 50 ml YPD medium, 28 °C) were used to inoculate fermentation at a density of 106 cells/ml. Fermentation was carried out in synthetic MS medium, which contained 240 g/L sugars (equimolar mixture of glucose and fructose), 6 g/L malic acid, 6 g/L citric acid and 200 mg/L nitrogen in the form of amino acids (148 mg N/L) and NH4Cl (52 mg N/L), at pH 3.5 (5). Ergosterol (1.875 mg/L), oleic acid (0.625 mg/L) and Tween 80 (0.05 g/L) were provided as anaerobic growth factors. Fermentation took place in 1.1-liter fermentors equipped with fermentation locks to maintain anaerobiosis, at 28 °C, with continuous magnetic stirring (500 rpm). CO_2_ release was followed by automatic measurements of fermentor weight loss every 20 min. The amount of CO_2_ released allowed us to monitor the progress of the fermentation. Samples were harvested for further analysis when the released CO_2_ reached approximately 11 g. The dry weight of the yeast was measured by filtering 50 mL of culture through a 0.45-mm-pore Millipore nitrocellulose filter, which was washed twice with 50 mL distilled water and dried for 24 h at 105 °C. Metabolites in the supernatant (acetate, succinate, glycerol, alpha-ketoglutarate, pyruvate and ethanol) were analyzed by high-pressure liquid chromatography [36]. Acetaldehyde production was determined with an enzymatic UV method [58].

Fermentation was carried out in duplicate spread over various fermentation blocks. Data (six metabolites, biomass) were first normalized by the released CO_2_. We then used a linear mixed model (Rstudio, nlme package) to correct measures for “block” effects, and the average values between the two replicates were calculated. From these normalized and corrected data, we recalculated the biomass and metabolite concentrations corresponding to 11 g/L of CO_2_.

### Model

Metabolite concentrations (in mmol ml^−1^) and dry weight (g L^−1^) were used to constrain DynamoYeast, a previously developed dedicated constraint-based model of yeast fermentative central carbon metabolism [9]. This model is composed of three compartments: the cytoplasm, mitochondria and extracellular medium, and includes 61 metabolites (Additional file 2: Table S2 for full name and abbreviations) and 68 reactions (Additional file 2: Table S3). For each of the 43 strains, we used the corrected metabolite concentrations to constrain the corresponding output flux of the model and the measured dry weight to constrain the flux of biomass (Additional file 2: Table S1). We used the experimental measures +2.5 and −2.5 % at the upper and lower flux boundaries, respectively. Then, we performed a flux balance analysis (FBA) minimizing the flux of glucose input (Glc_t) to obtain the flux distribution through the metabolic network [9]. In contrast to other standard constraint-based methods that compute flux distribution based on the derivation of mass data, here we directly computed mass distribution, as in Celton et al. [9].

We considered that all sugars were glucose (instead of glucose and fructose) for the modeling approach, as this assumption did not impact the flux predictions. For all strains, we used the biomass composition previously determined for the EC1118 strain [9] and set the cytosolic isocitrate dehydrogenase reaction (*IDP2*, YLR174W), the mitochondrial glutamate dehydrogenase reaction (*GDH2*, YDL215C) and the futile cycle around glycerol [9] to 0.

All predictions were performed with Matlab R2010b. The flux balance analysis (FBA) was performed with the “optimizeCbModel” function from the cobra toolbox [59] and the GLPK solver. The evaluation of the number of alternative solutions was done with the “enumerateOptimalSolution” algorithm [40] from a model where all input and output fluxes had been constrained by their exact predicted value from the FBA optimization.

### Statistical analysis

For each strain, we obtained a prediction of the flux distribution through the metabolic network. However, the predicted glucose uptake was different for each strain. To compare flux distributions between strains, we normalized each flux to the specific glucose uptake in the corresponding strain and expressed it as a percentage. We calculated the coefficient of variation for each flux: the standard deviation divided by the mean flux of all strains.

On a subset of 19 fluxes, we calculated the relative deviation from the average $\left( {\frac{{Flux_{i} - Flux_{mean} }}{{Flux_{mean} }}} \right),$ which gave an idea of how far a given strain was from the average distribution. To analyze the effect of strain origin on selected relative deviations, we used a linear model with a fixed effect of origins and ANOVA.

Principal component analysis of flux values was performed with fourteen fluxes that were representative of the entire model’s network, with the exception of the glycolysis and ethanol synthesis fluxes. All analysis and graphical representations were performed with RStudio [60] and with the following packages: “FactoMineR,” “corrplot,” “gplots” and “XML.” The graphical representations were later modified with Inckscape (http://www.inkscape.org) for visual ameliorations.

## Additional files

10.1186/s12934-016-0456-0 **Table S1**: Strains' origin and experimental data. **Table S2**: Model’s metabolites abbreviations. **Table S3**: Model’s reactions.
10.1186/s12934-016-0456-0 **Fig. S1**: Proportion of the NAPDH demand produced by the PPP and the acetate synthesis for each strain. Proportion of the NAPDH demand produced by the PPP (red) and the acetate synthesis (green) represented for each strain as a vertical bar. The horizontal black line represents the average proportion of the NAPDH demand produced by the PPP. **Fig. S2**: Comparison of the second and third axis of PCA made from the fluxes or the biological data. Graphical representation of strain fluxes projected on the two plans defined by the second and third axes of the PCA. The strains are represented as dots colored in function of strain origin. On top of each graph is the circle of variables. PCA calculated from 14 predicted fluxes (a). PCA calculated from 7 experimental data (b).

## Abbreviations

CBM: constraint-based model
MFA: metabolic flux analysis
FBA: flux balance analysis
PPP: pentose phosphate pathway
CCM: central carbon metabolism
E4P: erythrose-4-phosphate
R5p: ribose-5-phosphate
AKG: alpha-ketoglutarate
