# Supplementary material for: Diversity of flux distribution in central carbon metabolism of S. cerevisiae strains from diverse environments
Source: Microb Cell Fact. 2016 Apr 5;15:58. doi: 10.1186/s12934-016-0456-0 (PMC4820951; doi:10.1186/s12934-016-0456-0)
Supplement: Supplementary file 1 — 10.1186/s12934-016-0456-0 Table S1: Strains' origin and experimental data. Table S2: Model’s metabolites abbreviations. Table S3: Model’s reactions. [file 12934_2016_456_MOESM1_ESM.docx]

| **Additional Table S1: Strains' origin and experimental data** | | |  |  |  |  |  |  |  |  |
| --- | --- | --- | --- | --- | --- | --- | --- | --- | --- | --- |
|  |  |  |  | | | | | |  |  |
| Name | Ecological Origin* | Origin^#^ | Succinate^1^ | Glycerol^1^ | Acetate^1^ | Pyruvate^1^ | AKG^1^ | Ethanol^1^ |  | Biomass^2^ |
| 6464 | Bread | Bread, Japan (Lallemand), Asia | 2.29 | 27.04 | 2.31 | 0.87 | 0.20 | 276.56 |  | 1.24 |
| CBS1171 | Bread | Beer, neotype of Saccharomyces cerevisiae | 1.58 | 26.51 | 1.47 | 0.99 | 0.13 | 256.72 |  | 1.66 |
| CLIB215 | Bread | Bread, New Zealand, Oceania | 2.34 | 26.00 | 0.98 | 1.01 | 0.28 | 270.48 |  | 2.02 |
| CLIB215_3B | Bread | Bread, derivative spore from CLIB215, New Zealand, Oceania | 2.56 | 29.66 | 1.92 | 1.17 | 0.26 | 291.76 |  | 1.91 |
| 7_7 | Flor | Flor, natural diploid flor yeast isolated in Spain, Europe | 1.54 | 23.92 | 6.53 | 0.56 | 0.15 | 272.35 |  | 1.44 |
| F25 | Flor | Flor, natural diploid flor yeast isolated in Cordoba, Spain, Europe | 0.87 | 22.68 | 6.38 | 0.49 | 0.11 | 251.43 |  | 1.22 |
| FS2D | Flor | Flor, spore from a flor yeast isolated in Sardinia, Italy, Europe | 1.47 | 24.21 | 6.63 | 0.68 | 0.24 | 277.12 |  | 1.12 |
| GUF54_A1 | Flor | Flor, derivative spore from a flor yeast isolated in Jura, France, Europe | 1.87 | 23.48 | 6.77 | 1.03 | 0.26 | 280.77 |  | 1.07 |
| MJ73 | Flor | Wine, Lebanon, Asia | 1.63 | 23.37 | 6.02 | 0.82 | 0.32 | 272.92 |  | 1.63 |
| P3_D5 | Flor | Flor, derivative spore from a flor yeast isolated in Jura, France, Europe | 1.49 | 20.48 | 4.77 | 0.85 | 0.19 | 272.30 |  | 1.26 |
| TA12_2 | Flor | Flor, derivative spore from a flor yeast isolated in Hungary, Europe | 1.66 | 22.88 | 4.76 | 0.96 | 0.27 | 277.92 |  | 1.50 |
| TS12_A7 | Flor | Flor, natural flor yeast isolated in Hungary, Europe | 3.03 | 21.97 | 5.70 | 0.72 | 0.24 | 268.66 |  | 1.71 |
| VPDN_Fino | Flor | Flor, velum isolate of Flor ageing wine, Spain, Europe | 3.24 | 21.63 | 3.23 | 0.84 | 0.26 | 269.14 |  | 1.13 |
| OakR3 | MedOak | Oak, Romania, Europe | 1.69 | 27.16 | 4.42 | 1.05 | 0.17 | 293.53 |  | 1.60 |
| ZP848 | MedOak | Oak (Quercus ilex), Alter do Chão, Portugal, Europe | 1.88 | 26.75 | 4.75 | 1.04 | 0.16 | 277.65 |  | 2.09 |
| ZP851 | MedOak | Oak (Quercus ilex) Alcornocales Natural Park, Andalusia, Spain, Europe | 1.75 | 26.54 | 3.56 | 1.02 | 0.16 | 276.84 |  | 1.68 |
| OakA11 | Oak | Oak, Ardèche, France, Europe | 1.36 | 28.64 | 7.12 | 1.00 | 0.12 | 279.12 |  | 2.11 |
| OakB21 | Oak | Oak, Bordeaux, Gironde, France, Europe | 1.45 | 27.38 | 6.74 | 1.00 | 0.12 | 270.88 |  | 2.13 |
| ZP1050 | Oak | Oak (Quercus ilex), Reguengos de Monsaraz, Beja, Portugal, Europe | 1.93 | 27.34 | 5.54 | 0.92 | 0.16 | 297.84 |  | 1.71 |
| ZP611 | Oak | Oak (Quercus robur), Vancouver, Canada, North America | 1.85 | 26.96 | 5.03 | 0.97 | 0.26 | 291.79 |  | 2.08 |
| 245 | Rum | Rum distillery, French West Indies, North America | 1.59 | 24.68 | 4.08 | 0.63 | 0.17 | 257.83 |  | 1.97 |
| 309 | Rum | Rum distillery, French West Indies, North America | 2.16 | 26.71 | 4.36 | 0.80 | 0.30 | 278.00 |  | 1.87 |
| 376 | Rum | Rum distillery, French West Indies, North America | 1.92 | 23.13 | 4.84 | 0.75 | 0.15 | 278.53 |  | 2.32 |
| 460 | Rum | Rum distillery, French West Indies, North America | 2.08 | 23.94 | 3.93 | 0.97 | 0.27 | 277.96 |  | 1.97 |
| 390_D2 | Rum | Rum distillery, French West Indies derivative spore from strain 390, North America | 2.05 | 24.37 | 3.95 | 0.94 | 0.21 | 276.10 |  | 1.38 |
| CBS7957 | Rum | Factory producing cassava flour, Brazil, South America | 1.79 | 22.50 | 3.70 | 0.91 | 0.15 | 271.30 |  | 1.70 |
| CBS7959 | Rum | Factory producing fuel ethanol from sugar cane, Brazil | 1.68 | 22.63 | 4.75 | 0.98 | 0.19 | 282.28 |  | 1.35 |
| EDV493 | Rum | Rum distillery, French West Indies, North America | 2.11 | 21.82 | 3.17 | 0.76 | 0.15 | 277.20 |  | 1.66 |
| 1014_F5 | Wine | Wine, derivative spore F5 from the wine strain 1014, Italy, France, Europe | 2.15 | 25.21 | 3.24 | 1.33 | 0.49 | 285.37 |  | 1.22 |
| 20B2 | Wine | Wine, derivative spore from the wine strain L2056 isolated in Côtes du Rhône, France, Europe | 1.76 | 21.68 | 2.47 | 0.91 | 0.40 | 272.31 |  | 1.71 |
| 22A4 | Wine | Wine, derivative spore from the wine strain L2226 isolated in Côtes du Rhône, France, Europe | 1.89 | 21.66 | 2.57 | 0.82 | 0.46 | 253.04 |  | 1.73 |
| 6320_A7 | Wine | Wine, derivative spore from the wine strain, Italy, Europe | 1.94 | 23.27 | 3.68 | 1.06 | 0.44 | 273.04 |  | 2.21 |
| D47_6 | Wine | Wine, derivative spore from the wine strain D47, Europe | 2.03 | 23.18 | 1.88 | 0.76 | 0.20 | 324.46 |  | 1.87 |
| EC1118 | Wine | Wine (commercial), France, Europe | 1.83 | 22.90 | 3.95 | 0.81 | 0.15 | 295.43 |  | 2.20 |
| F12_3B | Wine | Flor, natural diploid flor yeast isolated in Cordoba, Spain, Europe | 2.23 | 24.77 | 2.96 | 0.86 | 0.23 | 287.49 |  | 1.95 |
| GE7_4A | Wine | Flor, derivative spore from the flor yeast GE7 isolated in Alsace, France, Europe | 1.97 | 22.07 | 2.31 | 0.84 | 0.22 | 309.87 |  | 1.44 |
| K1_28_1A | Wine | Wine, derivative spore from the wine strain K1, France, europe | 2.37 | 21.58 | 2.05 | 0.83 | 0.39 | 282.86 |  | 1.34 |
| L1414 | Wine | Wine, natural diploid wine yeast isolated in Beaujolais, France, Europe | 1.66 | 22.15 | 3.39 | 0.86 | 0.36 | 292.66 |  | 1.94 |
| Lava32_15 | Wine | Grape (Vitis vinifera) isolate of an abandoned vineyard, Azores, Portugal, Europe | 1.88 | 26.35 | 4.02 | 0.87 | 0.23 | 301.04 |  | 2.00 |
| Lava32_6 | Wine | Grape (Vitis vinifera) isolate of an abandoned vineyard, Azores, Portugal, Europe | 1.77 | 22.99 | 3.23 | 0.86 | 0.30 | 284.45 |  | 1.61 |
| M15-3B | Wine | Grape (Vitis vinifera) isolate, Montpellier, France , Europe | 1.89 | 21.88 | 3.61 | 0.79 | 0.20 | 295.00 |  | 1.46 |
| MC10 | Wine | Grape (Vitis vinifera) isolate, Montpellier, France , Europe | 1.85 | 22.22 | 3.49 | 0.96 | 0.31 | 289.20 |  | 1.62 |
| MC3C | Wine | Grape (Vitis vinifera) isolate, Montpellier, France , Europe | 2.06 | 22.84 | 3.11 | 1.08 | 0.39 | 298.98 |  | 2.00 |
| MO1A | Wine | Grape (Vitis vinifera) isolate, Montpellier, France , Europe | 2.09 | 22.12 | 2.79 | 0.92 | 0.25 | 305.80 |  | 2.68 |
| N15_4 | Wine | Wine, Moldavia, Romania, europe | 1.91 | 21.43 | 1.20 | 0.76 | 0.35 | 280.96 |  | 1.72 |
|  |  |  |  |  |  |  |  |  |  |  |
| *Strain's origins used in this study | |  |  |  |  |  |  |  |  |  |
| ^#^Geographical location of their isolation. | | |  |  |  |  |  |  |  |  |
| ^1^ Metabolite concentration in mmol.L^-1^ | | |  |  |  |  |  |  |  |  |
| ^2^ Biomass concentration in g.L^-1^ | | |  |  |  |  |  |  |  |  |
|  |  |  |  |  |  |  |  |  |  |  |
|  |  |  |  |  |  |  |  |  |  |  |
|  |  |  |  |  |  |  |  |  |  |  |

**Additional Table S2: Model’s metabolites abbreviations**

| Metabolite abbreviation | Compartments | Metabolite description |
| --- | --- | --- |
| 13dpg[c] | cytoplasm | 3_Phospho_D_glyceroyl_phosphate |
| 2pg[c] | cytoplasm | D_Glycerate_2_phosphate |
| 3pg[c] | cytoplasm | 3_Phospho_D_glycerate |
| 6pgc[c] | cytoplasm | 6_Phospho_D_gluconate |
| 6pgl[c] | cytoplasm | 6_phospho_D_glucono_1_5_lactone |
| ac[c] | cytoplasm | Acetate |
| ac[m] | mitochondria | Acetate |
| acald[c] | cytoplasm | Acetaldehyde |
| acald[m] | mitochondria | Acetaldehyde |
| accoa[c] | cytoplasm | Acetyl_CoA |
| accoa[m] | mitochondria | Acetyl_CoA |
| adp[c] | cytoplasm | ADP |
| adp[m] | mitochondria | ADP |
| akg[c] | cytoplasm | Alpha ketoglutarate |
| akg[m] | mitochondria | Alpha ketoglutarate |
| amp[m] | mitochondria | AMP |
| atp[c] | cytoplasm | ATP |
| atp[m] | mitochondria | ATP |
| cit[m] | mitochondria | Citrate |
| co2[c] | cytoplasm | CO2 |
| co2[m] | mitochondria | CO2 |
| coa[c] | cytoplasm | Coenzyme_A |
| coa[m] | mitochondria | Coenzyme_A |
| dhap[c] | cytoplasm | Dihydroxyacetone_phosphate |
| e4p[c] | cytoplasm | D_Erythrose_4_phosphate |
| etoh[c] | cytoplasm | Ethanol |
| etoh[m] | mitochondria | Ethanol |
| f6p[c] | cytoplasm | D_Fructose_6_phosphate |
| fdp[c] | cytoplasm | D_Fructose_1_6_bisphosphate |
| fum[c] | cytoplasm | Fumarate |
| fum[m] | mitochondria | Fumarate |
| g3p[c] | cytoplasm | Glyceraldehyde_3_phosphate |
| g6p[c] | cytoplasm | D_Glucose_6_phosphate |
| glc[c] | cytoplasm | D_Glucose |
| gln[c] | cytoplasm | L_Glutamine |
| glu[c] | cytoplasm | L_Glutamate |
| glu[m] | mitochondria | L_Glutamate |
| glyc[c] | cytoplasm | Glycerol |
| glyc3p[c] | cytoplasm | Glycerol_3_phosphate |
| icit[m] | mitochondria | Isocitrate |
| mal[c] | cytoplasm | L_Malate |
| mal[m] | mitochondria | L_Malate |
| nad[c] | cytoplasm | Nicotinamide_adenine_dinucleotide |
| nad[m] | mitochondria | Nicotinamide_adenine_dinucleotide |
| nadh[c] | cytoplasm | Nicotinamide_adenine_dinucleotide_reduced |
| nadh[m] | mitochondria | Nicotinamide_adenine_dinucleotide_reduced |
| nadp[c] | cytoplasm | Nicotinamide_adenine_dinucleotide_phosphate |
| nadp[m] | mitochondria | Nicotinamide_adenine_dinucleotide_phosphate |
| nadph[c] | cytoplasm | Nicotinamide_adenine_dinucleotide_phosphate_reduced |
| nadph[m] | mitochondria | Nicotinamide_adenine_dinucleotide_phosphate_reduced |
| oaa[c] | cytoplasm | Oxaloacetate |
| oaa[m] | mitochondria | Oxaloacetate |
| pyr[c] | cytoplasm | Pyruvate |
| pyr[m] | mitochondria | Pyruvate |
| r5p[c] | cytoplasm | alpha_D_Ribose_5_phosphate |
| ru5p[c] | cytoplasm | D_Ribulose_5_phosphate |
| s7p[c] | cytoplasm | Sedoheptulose_7_phosphate |
| succ[c] | cytoplasm | Succinate |
| succ[m] | mitochondria | Succinate |
| succoa[m] | mitochondria | Succinyl_CoA |
| xu5p[c] | cytoplasm | D_Xylulose_5_phosphate |

**Additional Table S3: Model’s reactions**

Abbrevation* Compartments Reaction

Glc_G6p cytoplasm glc[c] + atp[c] --> g6p[c] + adp[c]

G6p_F6p cytoplasm g6p[c] <==> f6p[c]

F6p_Fdp cytoplasm f6p[c] + atp[c] --> fdp[c] + adp[c]

Fdp_Dhap cytoplasm fdp[c] <==> dhap[c] + g3p[c]

Dhap_G3p cytoplasm dhap[c] <==> g3p[c]

G3p_13dpg cytoplasm g3p[c] + nad[c] <==> 13dpg[c] + nadh[c]

13dpg_3pg cytoplasm 13dpg[c] + adp[c] <==> 3pg[c] + atp[c]

3pg_2pg cytoplasm 3pg[c] <==> 2pg[c]

2pg_Pep cytoplasm 2pg[c] <==> pep[c]

Pep_Pyr cytoplasm pep[c] + adp[c] --> pyr[c] + atp[c]

G6p_6pgl cytoplasm g6p[c] + nadp[c] <==> 6pgl[c] + nadph[c]

6pgl_6pgc cytoplasm 6pgl[c] --> 6pgc[c]

6pgc_Ru5p cytoplasm 6pgc[c] + nadp[c] --> co2[c] + nadph[c] + ru5p[c]

Ru5p_Xu5p cytoplasm ru5p[c] <==> xu5p[c]

Ru5p_R5p cytoplasm ru5p[c] <==> r5p[c]

R5p_S7p cytoplasm r5p[c] + xu5p[c] <==> g3p[c] + s7p[c]

E4p_F6p cytoplasm e4p[c] + xu5p[c] <==> f6p[c] + g3p[c]

S7p_E4p cytoplasm g3p[c] + s7p[c] <==> e4p[c] + f6p[c]

Dhap_Glyc3p cytoplasm dhap[c] + nadh[c] --> glyc3p[c] + nad[c]

Glyc3p_Glyc cytoplasm glyc3p[c] --> glyc[c]

Pyr_Acald cytoplasm pyr[c] --> acald[c] + co2[c]

Acald_Eth cytoplasm acald[c] + nadh[c] --> etoh[c] + nad[c]

Acald_Ac cytoplasm acald[c] + nadp[c] --> ac[c] + nadph[c]

Ac_Accoa cytoplasm ac[c] + 2 atp[c] --> accoa[c] + 2 adp[c]

Pyr_Oaa cytoplasm pyr[c] + atp[c] + co2[c] --> oaa[c] + adp[c]

Acald_Eth_m mitochondria acald[m] + nadh[m] <==> etoh[m] + nad[m]

Acald_Ac_m mitochondria acald[m] + nadp[m] --> ac[m] + nadph[m]

Oaa_Mal cytoplasm oaa[c] + nadh[c] <==> mal[c] + nad[c]

Mal_Fum cytoplasm mal[c] --> fum[c]

Fum_Succ cytoplasm fum[c] --> succ[c]

Akg_Glu cytoplasm akg[c] + nadph[c] <==> glu[c] + nadp[c]

Glu_Akg_m mitochondria glu[m] + nad[m] --> akg[m] + nadh[m]

Pyr_Accoa_m mitochondria pyr[m] + nad[m] --> accoa[m] + nadh[m] + co2[m]

Oaa_Cit_m mitochondria accoa[m] + oaa[m] --> cit[m]

Cit_Icit_m mitochondria cit[m] <==> icit[m]

Icit_Akg_m_nad mitochondria icit[m] + nad[m] --> akg [m]+ co2[m] + nadh[m]

Icit_Akg_m_nadp mitochondria icit[m] + nadp[m] --> akg[m] + co2[m] + nadph[m]

Akg_Succoa_m mitochondria akg[m] + nad[m] --> succoa[m] + co2[m] + nadh[m]

Succoa_Succ_m mitochondria succoa[m] + adp[m] --> succ[m] + atp[m]

Oaa_Mal_m mitochondria oaa[m] + nadh[m] --> mal[m] + nad[m]

Mal_Fum_m mitochondria mal[m] --> fum[m]

Fum_Succ_m mitochondria fum[m] --> succ[m]

Mal_Pyr_m mitochondria mal[m] + nadp[m] --> co2[m] + nadph[m] + pyr[m]

Acald_tm transport acald[c] <==> acald[m]

Succ_tm transport succ[c] + atp[c] --> succ[m] + adp[c]

Mal_tm transport mal[c] + atp[c] --> mal[m] + adp[c]

Mal_Succ_tm transport mal[c] + succ[m] <==> mal[m] + succ[c]

Pyr_tm transport pyr[c] + atp[c] --> pyr[m] + adp[c]

Akg_tm transport akg[c] <==> akg[m]

Oaa_tm transport oaa[c] + atp[c] --> oaa[m] + adp[c]

Eth_tm transport etoh[c] <==> etoh[m]

CO2_tm transport co2[c] <==> co2[m]

Ac_tm transport ac[c] <==> ac[m]

Accoa_tm transport accoa[c] --> accoa[m]

Glu_tm transport glu[c] + atp[c] --> glu[m] + adp[c]

Glc_t transport glc[c] -->

Eth_t transport etoh[c] -->

Ac_t transport ac[c] -->

Pyr_t transport pyr[c] -->

Akg_t transport akg[c] -->

Succ_t transport succ[c] -->

But_t transport but[c] -->

Aceto_t transport aceto[c] -->

Acald_t transport acald[c] -->

Glyc_t transport glyc[c] -->

CO2_t transport co2[c] <==>

ATP_Shuttle cytoplasm atp <==> adp

BIOMASS biomass 3.96 g6p[c] + 0.258 r5p[c] + 0.129 e4p[c] + 0.116 g3p[c] + 0.303 3pg[c] + 0.232 pep[c] + 0.775 oaa[c] + 1.084 pyr[m] + 0 pyr[c] + 0.176 accoa[m] + 0.252 accoa[c] + 0.106 akg[m] + 0.366 akg[c] + 0 co2[c] + 0.136 glu[c] + 115 atp[c] + 0.106 atp[m] + 1.499 nad[c] + 0.176 nad[m] + 0.602 nadph[m] + 5.35 nadph[c] --> 115 adp[c] + 0.106 adp[m] + 1.499 nadh[c] + 0.176 nadh[m] + 0.602 nadp[m] + 5.35 nadp[c]

* the abbreviation names are encoded as abbreviation of the substrate and the product connected by a “_”.

For mitochondrial reactions we added a “_m”.

Extracellular transport and mitochondrial transport reaction are encoded as the metabolic name abbreviation followed respectively by a “_t” and a “_tm”.

Metabolite abbreviations can be found in the spplumentary table 2.
