# Supplementary material for: Diversity of flux distribution in central carbon metabolism of S. cerevisiae strains from diverse environments
Source: Microb Cell Fact. 2016 Apr 5;15:58. doi: 10.1186/s12934-016-0456-0 (PMC4820951; doi:10.1186/s12934-016-0456-0)
Supplement: Supplementary file 2 — 10.1186/s12934-016-0456-0 Fig. S1: Proportion of the NAPDH demand produced by the PPP and the acetate synthesis for each strain. Proportion of the NAPDH demand produced by the PPP (red) and the acetate synthesis (green) represented for each strain as a vertical bar. The horizontal black line represents the average proportion of the NAPDH demand produced by the PPP. Fig. S2: Comparison of the second and third axis of PCA made from the fluxes or the biological data. Graphical representation of strain fluxes projected on the two plans defined by the second and third axes of the PCA. The strains are represented as dots colored in function of strain origin. On top of each graph is the circle of variables. PCA calculated from 14 predicted fluxes (a). PCA calculated from 7 experimental data (b). [file 12934_2016_456_MOESM2_ESM.pdf]

Figure S1

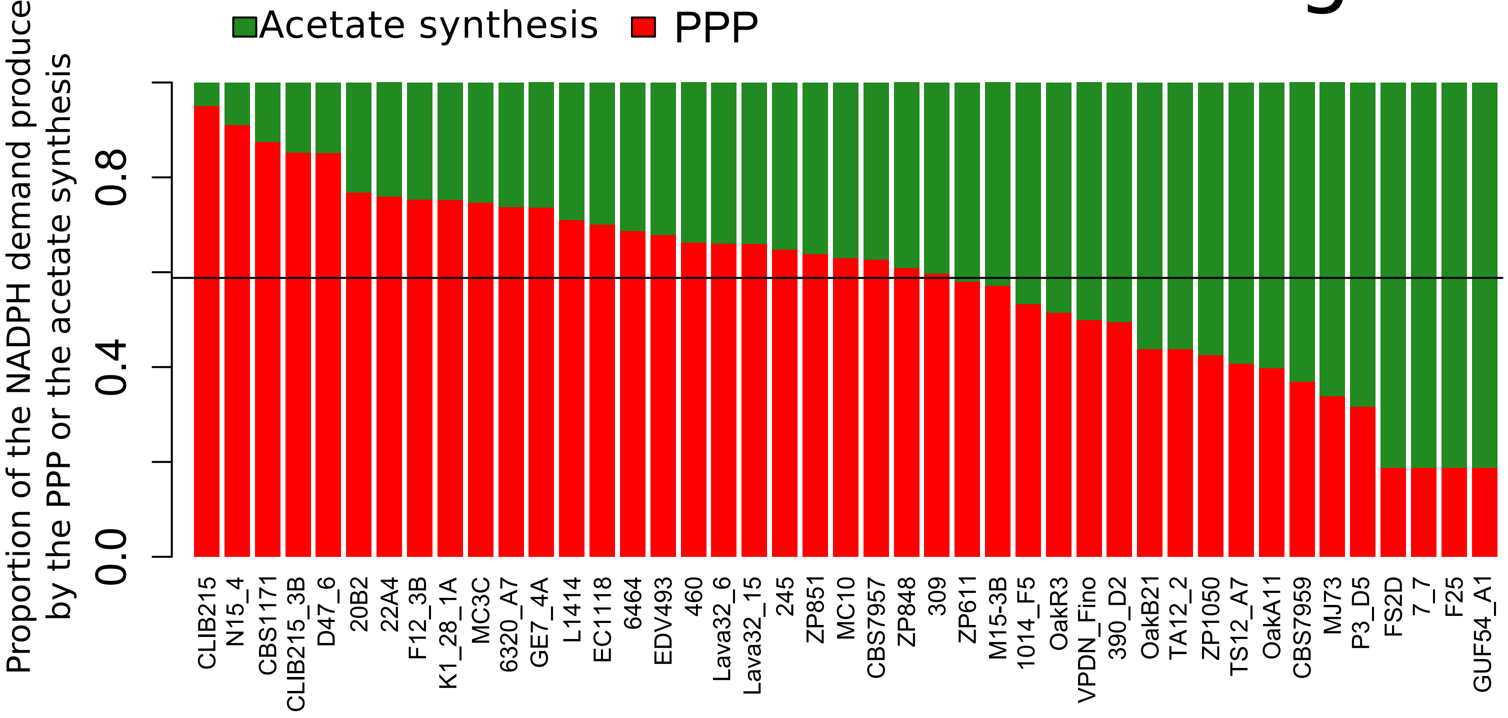

Acetate synthesis
PPP
Proportion of the NADPH demand produce
by the PPP or the acetate synthesis
0.0
0.4
0.8
CLIB215
N15_4
CBS1171
CLIB215_3B
D47_6
20B2
22A4
F12_3B
K1_28_1A
MC3C
6320_A7
GE7_4A
L1414
EC1118
6464
EDV493
460
Lava32_6
Lava32_15
245
ZP851
MC10
CBS7957
ZP848
309
ZP611
M15-3B
1014_F5
OakR3
VPDN_Fino
390_D2
OakB21
TA12_2
ZP1050
TS12_A7
OakA11
CBS7959
MJ73
P3_D5
FS2D
7_7
F25
GUF54_A1

Figure S2

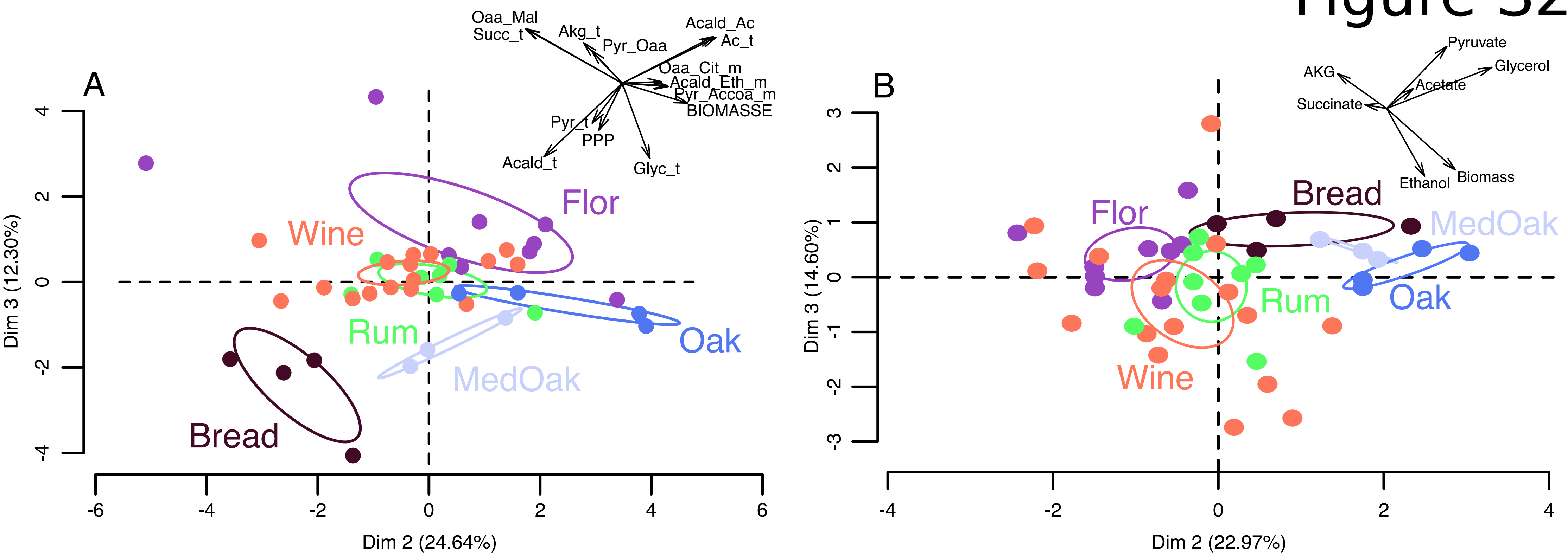
A
Oaa_Mal
Succ_t
Akg_t
Pyr_Oaa
Acald_Ac
Ac_t
Oaa_Cit_m
Acald_Eth_m
Pyr_Accoa_m
BIOMASSE
Pyr_t
PPP
Acald_t
Glyc_t
Flor
Wine
Rum
Oak
MedOak
Bread
Dim 3 (12.30%)
Dim 2 (24.64%)
4
2
0
-2
-4
-6
6
B
Pyruvate
Glycerol
AKG
Acetate
Succinate
Ethanol
Biomass
Bread
MedOak
Flor
Rum
Oak
Wine
Dim 3 (14.60%)
Dim 2 (22.97%)
3
2
1
0
-1
-2
-3
-4
4

Fig. S1: Proportion of the NAPDH demand produce by both the PPP and the acetate synthesis for each strain.

Proportion of the NAPDH demand produced by the PPP (red) and the acetate synthesis (green) represented for each strain as a vertical bar. The horizontal black line represents the average proportion of the NAPDH demand produced by the PPP.

Fig. S2: Comparison of the second and third axis of PCA made from the fluxes or the biological data.

Graphical representation of strain fluxes projected on the two plans defined by the second and third axes of the PCA. The strains are represented as dots colored in function of strain origin. On top of each graph is the circle of variables. PCA calculated from 14 predicted fluxes (a). PCA calculated from 7 experimental data (b).
